# Control and determinants of the tobacco epidemic and the use of other tobacco products among children and adolescents in the Subcarpathian Voivodeship

**DOI:** 10.3389/fpubh.2025.1629481

**Published:** 2025-10-02

**Authors:** Paulina Hejda, Artur Mazur, Julia Trojniak, Sara Bień, Marta Kopańska

**Affiliations:** ^1^Faculty of Health Sciences and Psychology, University of Rzeszów, Rzeszow, Poland; ^2^Faculty of Medicine, University of Rzeszów, Rzeszow, Poland; ^3^Student Research Club “Reh-Tech”, Faculty of Medicine, University of Rzeszów, Rzeszow, Poland; ^4^Department of Medical Psychology, Faculty of Medicine, University of Rzeszów, Rzeszow, Poland

**Keywords:** tobacco smoking, children, adolescents, cigarettes, e-cigarettes, prevention, public health, tobacco

## Abstract

**Introduction:**

Tobacco smoking remains one of the most significant public health challenges, particularly among children and adolescents, for whom early smoking initiation increases the risk of long-term addiction and severe health consequences. The aim of this study was to assess the prevalence of cigarette and other tobacco product use among adolescents aged 12–16 years in the Subcarpathian Voivodeship and to analyze the factors influencing this phenomenon.

**Materials and method:**

The study involved a survey conducted among 865 students aged 12–16 years from the Subcarpathian Voivodeship. The research tool was a questionnaire comprising 79 questions addressing nicotine initiation, tobacco product availability, and peer behaviors. Data were collected between March and November 2019. Educational institutions were randomly selected, and parents or legal guardians were informed via an electronic journal. The study was conducted with the consent of the Subcarpathian Education Authority and the Bioethics Committee of the University of Rzeszow.

**Results:**

The results indicated that 19.2% of respondents had experimented with cigarettes, with an average initiation age of 14 years. Notably, 31.1% of participants perceived e-cigarettes as less harmful than traditional cigarettes, highlighting the need for more intensive health education efforts. Most respondents reported smoking initiation due to peer influence, and some were able to purchase cigarettes independently, despite legal restrictions.

**Conclusion:**

The findings underscore the urgent need to intensify preventive measures, strengthen health education, and enhance enforcement of tobacco sales regulations to effectively curb the tobacco epidemic among the youngest populations.

**Implications:**

The study provides valuable data on the factors influencing tobacco and other tobacco product use among children and adolescents in the Subcarpathian Voivodeship. The findings highlight the crucial role of the social environment, access to tobacco products, and the effectiveness of preventive measures. The obtained data may serve as a basis for developing more effective tobacco prevention strategies for young people, as well as for implementing educational programs and policy interventions aimed at reducing the availability and attractiveness of tobacco products.

## 1 Introduction and objective

According to World Health Organization (WHO) tobacco smoking is a global health issue, leading to disease and premature mortality. It is estimated to account for 12% of deaths among individuals over 30 years of age (1). Tobacco use poses a threat to both active and passive smokers. According to data, smoking is responsible for approximately 8 million deaths annually, including 1.2 million deaths among non-smokers exposed to second-hand smoke (2). The introduction of e-cigarettes and modern flavored tobacco products has exacerbated the problem among adolescents (3). E-cigarettes deliver nicotine through an aerosol produced by heating a liquid, often referred to as “vapor,” which in reality contains propylene glycol or glycerine, nicotine, and flavoring agents (4). These products, developed in the 21st century, were initially intended to aid smoking cessation but quickly gained popularity, with sales expanding through various online platforms (5). As early as 2014, Goniewicz et al. raised alarms by documenting a more than five-fold increase in current e-cigarette use among Polish adolescents to 29.9%, noting that traditional smoking also rose simultaneously (6). A 2024 study by Kurdyś-Bykowska et al., based on 2021 data, identified key demographic risk factors for e-cigarette use, including being male, living in a larger city, and attending a secondary technical school (7). The most recent 2022 Global Youth Tobacco Survey data, presented by Michalek et al., confirmed a “notably high” prevalence of 22.3% among 13–15 year old. This latest research also revealed a significant new trend, with usage being higher among girls (23.4%) than boys (21.2%) (8). These studies collectively illustrate a clear shift from traditional tobacco to a high prevalence of alternative nicotine products among Poland’s youth over the past decade.

In Poland, tobacco use is increasingly affecting younger age groups (4). Data show that over half of boys and girls aged 13–15 have attempted smoking, with 20% reporting initiation before the age of 10. The highest prevalence of e-cigarettes use among 13–15-year-olds in Europe is observed in Poland (23.4%), Ukraine (18.4%), Latvia (18.0%), and Italy (17.5%) (9).

Nicotine, the primary addictive component, increases the risk of numerous diseases, including cardiovascular disorders, heart attacks, impaired brain development in adolescents, and adverse fetal outcomes in pregnant women (10–12). Tobacco is one of the most significant risk factors for non-communicable diseases, such as lung cancer, chronic respiratory diseases, breast cancer, preterm birth, and pregnancy complications (13). Smoking also contributes to oral health issues, such as gum disease, tooth decay, and alterations in the oral microbiome (14). The diseases caused by tobacco smoke primarily result from the toxic effects of the substances it contains (15).

Given the significant issue of tobacco use among children and adolescents, the aim of this study was to evaluate the prevalence of smoking and the consumption of other tobacco products among school-aged youth, as well as an analysis of the impact of selected environmental factors on this phenomenon. The primary goal of this study was descriptive surveillance of adolescent tobacco use to inform local prevention efforts, not hypothesis testing or causal inference.

We hypothesized that the prevalence of tobacco use among adolescents aged 12–16 in the Subcarpathian Voivodeship is significant and is influenced by key sociodemographic factors such as peer influence, age, sex, and place of residence, as well as the perceived accessibility and harmfulness of tobacco products. The Subcarpathian Voivodeship is less urbanized and socioeconomically diverse, which may shape unique risk factors for tobacco use not captured in national surveys. Studying this population provides region-specific insights that complement national findings.

## 2 Materials and methods

### 2.1 Study design and recruitment

This cross-sectional survey was conducted from March to November 2019. Schools were randomly selected from 20 county towns. Principals were contacted with written invitations and information about study aims; 82% of approached schools agreed. Once schools consented, students were informed in classrooms and invited to participate. The target population was students aged 12–16 years. Of 865 invited, 771 provided complete data (89%).

The final sample consisted of 420 girls (54.5%) and 351 boys (45.5%). The average age of participants was 14.38 years (SD = 0.90). The majority of respondents (65.8%) lived in rural areas, 27.1% in small towns, and 7.1% in large cities.

#### 2.1.1 The main criteria adopted in the study

Inclusion criterion:

- age range from 12 to 16 years;
- verbally consent to participate in the study;
- return of the completed questionnaire.

Exclusion criterion:

- age under 12 years of age and over 16 years of age;
- lack of verbal consent to participate in the study;
- return of an incompletely completed questionnaire.

### 2.2 Participants

Complete questionnaires were obtained from 771 children and adolescents: 420 girls and 351 boys, representing 54.5 and 45.5% of the respondents, respectively. The average age of the participants was 14.38 ± 0.90 years. The sample consisted of students from grade seven of primary school (*n* = 370; 48.0%), as well as students from the second (*n* = 271; 35.1%) and third (*n* = 130; 16.9%) grades of the outgoing lower-secondary schools (gimnazja). The study was conducted in 2019, the final year of the *gimnazjum* system’s operation in Poland due to a nationwide educational reform, which explains the presence of students from both school types in the sample. The majority of respondents, 507 individuals (65.8%), indicated that they lived in rural areas, 209 (27.1%) in small towns, and 55 (7.1%) in large cities.

Of the 865 invited, 94 declined or returned incomplete surveys. The analytic sample of 771 students (420 girls, 351 boys) represented ~3.5% of the adolescent population of the voivodeship. Demographic data for the 94 individuals who declined to participate or returned incomplete surveys were not available, precluding a direct comparison between the included and excluded groups.

### 2.3 Ethical considerations

Parents and legal guardians were informed about the study’s objectives via electronic school journals, and their implied consent was obtained for their children’s participation. Students provided verbal consent to participate. The study was approved by the Subcarpathian Education Authority and the Bioethics Committee of the University of Rzeszow (Resolution No. 10/02/2019 dated February 14, 2019).

### 2.4 Data collection tool

The research tool was a Polish-language version of the Global Youth Tobacco Survey (GYTS) questionnaire, which has been validated for use in adolescent populations by the American Academy of Pediatrics (AAP). The validated Polish version of the GYTS questionnaire was administered during class hours in paper-and-pencil format, supervised by teachers. The self-administered questionnaire was anonymous and consisted of 79 questions divided into several categories:

- The first category included questions about age, sex, class, and place of residence (4 questions).
- The second category focused on thoughts about smoking, the desire to try smoking for the first time, offers to smoke with peers, age of smoking initiation, number of cigarettes smoked, brands of cigarettes, methods of obtaining cigarettes, and whether vendors refused to sell cigarettes (16 questions).
- The third section addressed access to and use of other tobacco products, as well as knowledge about various tobacco products such as roll-your-own cigarettes, bidi, kretek, hookah, water pipe, snuff, snus, and electronic cigarettes (22 questions).

### 2.5 Statistical analysis

Data analysis was conducted using descriptive and inferential statistics. Descriptive statistics, including frequencies (n) and percentages (%), were used to characterize the sample and summarize the prevalence of tobacco use. For inferential analysis, Pearson’s χ^2^ test was employed to examine relationships between categorical variables. In cases where expected cell counts were low, making the χ^2^ test imprecise, Fisher’s exact test was used instead. A two-proportion z-test was used to compare differences between two independent percentages. A *p*-value of <0.05 was considered statistically significant for all analyses. All calculations were performed using the STATISTICA 13 software package and Microsoft Excel. The data, being categorical, did not require tests for normal distribution. Because the original dataset is no longer accessible for re-analysis, all statistical procedures are limited to the existing descriptive and bivariate tests performed in 2019.

## 3 Results

Following the demographic items, the questionnaire addressed participants’ use of cigarettes and other tobacco products. Out of all respondents, 148 individuals (19.2%) admitted to smoking cigarettes, with 10% being primary school students and 27.7% middle school students, a statistically significant difference (*p* < 0.001). Among the respondents, 17.9% of girls and 20.8% of boys had attempted smoking. Those living in rural areas constituted 20.5%, while those in urban areas made up 16.7%. A total of 80.7% of respondents had never tried smoking cigarettes (Table 1).

**Table 1:** Initiation of tobacco smoking among respondents.

Characteristic			Have you ever tried smoking a cigarette, even one or two puffs?				
			Yes	No	No response	Total	*p*
Sex	Girls	n	75	344	1	420	0.393
		%	17.9	81.9	0.2	100	
	Boys	n	73	278	-	351	
		%	20.8	79.2	-	100	
School Level	Primary school (Grade 7)	n	37	333	-	370	< 0.01*
		%	10	90	-	100	
	Middle school	n	111	289	1	401	
		%	27.7	72.1	0.2	100	
Residence	Village	n	104	402	1	507	0.331
		%	20.5	79.3	0.2	100	
	City	n	44	220	-	264	
		%	16.7	83.3	-	100	
Total		n	148	622	1	771	
		%	19.2	80.7	0.1	100	

*Statistically significant.

This version accurately describes the central tendency for each group without making a confusing claim of a significant difference between them. Although the average age of smoking initiation was slightly lower for boys (13 years) compared to girls (14 years), this difference was not statistically significant (Table 2).

**Table 2:** The age of initiation of cigarette smoking by respondents.

Characteristic			How old were you when you first tried cigarettes, even one or two puffs?											
			8 years or less	9 years	10 years	11 years	12 years	13 years	14 years	15 years	16 years	No response	Total	*p*
Sex	Girls	n	4	3	2	6	15	14	19	12	-	-	75	0.198
		%	5.3	4	2.7	8	20	18.7	25.3	16	-	-	100	
	Boys	n	7	1	8	7	9	14	13	10	3	1	73	
		%	9.6	1.4	11	9.6	12.3	19.2	17.8	13.7	4.1	1.4	100	
School Level	Primary school (Grade 7)	n	2	1	4	9	5	11	4	-	-	1	37	*p* < 0.01*
		%	5.4	2.7	10.8	24.3	13.5	29.7	10.8	-	-	2.7	100	
	Lower-secondary school *(Gimnazjum)*	n	9	3	6	4	19	17	28	22	3	-	111	
		%	8.1	2.7	5.4	3.6	17.1	15.3	25.2	19.8	2.7	-	100	
Residence	Village	n	10	2	10	7	16	17	26	13	3	-	104	0.174
		%	9.6	1.9	9.6	6.7	15.4	16.3	25	12.5	2.9	-	100	
	City	n	1	2	-	6	8	11	6	9		1	44	
		%	2.3	4.5	-	13.6	18.2	25	13.6	20.5	-	2.3	100	
Total		n	11	4	10	13	24	28	32	22	3	1	148	
		%	7.4	2.7	6.8	8.8	16.2	18.9	21.6	14.9	2	0.7	100	

*Statistically significant.

The number of cigarettes smoked did not differ significantly by sex, educational level, or place of residence. The initial experience with smoking was limited to 1–2 puffs for 40% of girls and 27.4% of boys. Additionally, 22.3% of respondents smoked 2 to 5 cigarettes, while 18.7% of girls and 11% of boys reported smoking 6 to 15 cigarettes.

The survey also included questions about the brand of cigarettes smoked by children and adolescents in the Subcarpathian Voivodeship. The most frequently mentioned brand was L&M, cited by 30.8% of girls and 40% of boys. The second most common brand was Marlboro, mentioned by 19.6% of respondents. Notably, 42.3% of participants preferred menthol-flavored products, which have been withdrawn from the market.

An important aspect of the study was assessing the future smoking intentions of children and adolescents in the Subcarpathian Voivodeship. Regarding future smoking intentions, 43.6% of students (*n* = 337) denied any plan to smoke, 29.4% indicated they would ‘probably not’ smoke, and 7.0% expressed a definite intention to smoke.

The study also aimed to assess the knowledge of children and adolescents in the Subcarpathian Voivodeship regarding other tobacco products available on the market. These included roll-your-own cigarettes, hookahs, cigars, electronic cigarettes, snuff, and pipe tobacco. The results indicated that students were well-informed about these products, with 79.8% confirming not only their awareness but also their understanding of proper usage. Among the girls who acknowledged familiarity with other tobacco products, 14.4% smoked electronic cigarettes, and 6.4% used roll-your-own cigarettes. For boys, 21% smoked electronic cigarettes, and 9.8% used roll-your-own cigarettes, showing a statistically significant sex difference (*p* < 0.001). A month before the survey, 86.4% of respondents denied using tobacco products, considering only those who had previously declared usage.

An important aspect of the study was to determine how children and adolescents in the Subcarpathian Voivodeship obtained tobacco products. According to the law, these products should only be sold to individuals over 18 years of age. The most frequently reported method was receiving cigarettes from acquaintances (37.3%). Additionally, 17.6% of students managed to purchase cigarettes themselves, and 15.7% obtained them through older peers. However, there was no statistical significance difference for these data.

Opinions on the ease of purchasing cigarettes were varied: 47.2% of respondents indicated that it is not easy for minors, while 39.4% considered it ‘rather easy,’ and 10.0% stated that it poses no difficulty.

The study also highlighted the need for tobacco products among children and adolescents in the Subcarpathian Voivodeship. At the surveyed ages, nicotine addiction develops rapidly. A small but notable portion of students (8.3%) reported experiencing a craving and a need to smoke within the past month, whereas the vast majority (90.8%) denied any such desire.

To assess awareness of the harmful effects of smoking cigarettes and other tobacco products, respondents were asked about the consequences depicted on packaging illustrations. Among the respondents, 33.3% had not encountered a cigarette pack in the past month, 20.1% noticed warning signs on the illustrations each time they saw the packaging, and 14% usually saw them. These differences were statistically significant. Among primary (grade 7) and lower-secondary school *(gimnazjum)* students, 23.7% indicated that the illustrations depicting the consequences of smoking made them think.

Regarding the harmfulness of daily smoking, 74.2% of respondents provided answers, with 20.2% believing that cigarettes are somewhat harmful. These differences were statistically significant (Table 3).

**Table 3:** Respondents’ perception of the harm caused by daily smoking.

Characteristic			How much do people harm themselves if they smoke several cigarettes a day?						
			They do no harm	They do little harm	They do a little harm	They are very harmful	No response	Total	*p*
Sex	Girls	n	3	7	78	332	-	420	<0.001*
		%	0.7	1.7	18.6	79	-	100	
	Boys	n	16	14	78	240	3	351	
		%	4.6	4	22.2	68.4	0.9	100	
School Level	Primary school (Grade 7)	n	9	9	78	272	2	370	0.919
		%	2.4	2.4	21.1	73.5	0.5	100	
	Lower-secondary school *(Gimnazjum)*	n	10	12	78	300	1	401	
		%	2.5	3	19.5	74.8	0.2	100	
Residence	Village	n	15	13	91	388	-	507	0.015*
		%	3	2.6	17.9	76.5	-	100	
	City	n	4	8	65	184	3	264	
		%	1.5	3	24.6	69.7	1.1	100	
Total		n	19	21	156	572	3	771	
		%	2.5	2.7	20.2	74.2	0.4	100	

*Statistically significant.

The study also assessed knowledge regarding the harmfulness of other tobacco products, such as electronic cigarettes. Among the respondents, 31.3% believed that electronic cigarettes are less harmful than conventional cigarettes, while 22.4% lacked any knowledge on the subject (Table 4).

**Table 4:** Respondents’ knowledge about the harmfulness of electronic cigarettes.

Characteristic		Do you believe that electronic cigarettes or e-cigarettes are: (less harmful, just as harmful, more harmful) than regular cigarettes?								
		Less harmful	Just as harmful	More harmful		I have never tried an electronic or e-cigarette	I do not know enough about these products	No response	Total	*p*
Sex	Girls	n	103	90	29	102	96	-	420	<0.001*
		%	24.5	21.4	6.9	24.3	22.9	-	100	
	Boys	n	137	63	19	50	77	5	351	
		%	39	17.9	5.4	14.2	21.9	1.4	100	
School Level	Primary school (Grade 7)	n	109	65	24	72	97	3	370	0.192
		%	29.5	17.6	6.5	19.5	26.2	0.8	100	
	Lower-secondary school *(Gimnazjum)*	n	131	88	24	80	76	2	401	
		%	32.7	21.9	6	20	19	0.5	100	
Residence	Village	n	161	104	33	99	108	2	507	0.690
		%	31.8	20.5	6.5	19.5	21.3	0.4	100	
	City	n	79	49	15	53	65	3	264	
		%	29.9	18.6	5.7	20.1	24.6	1.1	100	
Total		n	240	153	48	152	173	5	771	
		%	31.1	19.8	6.2	19.7	22.4	0.6	100	

*Statistically significant.

The surveyed students believe that all tobacco products are harmful, with 89.5% expressing this view. Conversely, 6.2% denied any harmful effects, while the remaining students acknowledged the risks of smoking but did not consider all tobacco products to be harmful. Among the respondents, 42.4% believed that passive smoking is not detrimental to human health, whereas 41% considered it to be very harmful.

Given the significant influence of peers on children and adolescents, the study also examined whether smoking makes respondents feel more liked by others. The question was framed to address self-confidence, attractiveness, and the desire to be liked. Among the respondents, 71.2% did not believe that smoking makes them more liked by their peers, 15.8% answered “probably not,” and 3.5% felt more attractive when smoking. Additionally, respondents were asked whether smokers have more friends. Among the respondents, 51% stated that smoking does not affect their relationships, 25.8% answered “probably not,” and 20% believed that smoking helps in making friends.

The table presents a comparison of the study participants—those invited, included, and excluded—along with key sample characteristics (sex and place of residence). It also shows the total population of the Podkarpackie Voivodeship according to GUS data for 2019, providing a broader demographic context. The table highlights the proportion of respondents who completed the survey, the share of refusals or incomplete responses, and the internal structure of the study sample in terms of sex and residence type. Additionally, the authors’ note is included, indicating that the surveyed group represents approximately 3.5% of the adolescent population in the region (Table 5).

**Table 5:** Characteristics of invited, included, and excluded participants compared with the total population of Podkarpackie Voivodeship.

Category			Count (n)	% of invited (n/865)	Reference share
Sample characteristics	Sex	Girls	420	54.5% of included	~51%
		Boys	351	45.5% of included	~49%
	Residence	Rural residents	507	65.8% of included	~59%
		Urban residents (towns/cities)	264	34.2% of included	~41%
	Included (complete questionnaires)		771	89.1%	—
	Excluded (declined/incomplete)		94	10.9%	—
	Invited (total)		865	100.0%	—
Total population of Podkarpackie (GUS. 2019) (25)			2,127,200	—	100.0%
Share of adolescents covered			~3.5%	—	—

## 4 Discussion

Tobacco and its products are among the most well-known psychoactive substances globally. They are available in every country and cause stronger addiction than other products in this category. The widespread issue of tobacco smoking starkly contrasts with the harm and risks faced by both smokers and passive smokers. Nowadays, smoking has also become very popular among children and adolescents, who, observing adults, experiment without realizing the consequences of their actions. According to WHO data from a 2020 study, 22.3% of the global population over the age of 15 regularly used various forms of tobacco (16).

The study results indicate that smoking cigarettes and other tobacco products is a significant problem among children and adolescents in the Subcarpathian Voivodeship. Nicotine addiction is particularly dangerous at a young age, as it affects the development of the nervous system, increases the risk of cardiovascular diseases, and can lead to long-term health habits with negative consequences. Despite growing public awareness of nicotine’s harmful effects, 19.2% of respondents admitted to having contact with cigarettes, with the average age of initiation being 14 years. Alarmingly, 10% of children had their first contact with cigarettes at the age of 8–9 years. No level of exposure to cigarette smoke is safe, as even passive smoking is associated with a range of respiratory symptoms and serious diseases (17). This underscores the need for early health education and intensified preventive measures.

In a broader European context, our sample’s lifetime smoking prevalence (19.2%) is lower than the 25% average for 15-year-olds reported by the 2021/2022 HBSC survey. The trend is reversed for e-cigarettes, however. The HBSC report confirms their popularity has overtaken traditional cigarettes among adolescents (32% ever-use), with Poland ranking among the countries with the highest prevalence (18). On the other hand, our findings contrast with data from other parts of the world where the problem can be more severe. For instance, in a 2021 study of Mallol et al., involving 2,747 adolescents aged 13–15 years from low-income areas in Santiago de Chile, as many as 50.7% reported having ever tried smoking cigarettes (19). The absence of logistic regression or odds ratio estimation limits the ability to quantify associations; however, the large, randomly selected sample still provides reliable prevalence estimates that remain informative for regional policy.

The analysis showed no significant statistical differences between sex, educational level, and place of residence regarding the number of cigarettes smoked. Similar results were obtained in a 2021 study by Mallol et al., which also found no significant difference between girls and boys. The mentioned results further showed that 16.8% of respondents smoked an entire cigarette before the age of 12, and 62.3% were passive smokers at home (19). Additionally, girls were more likely to limit themselves to a few puffs, while boys were more inclined to smoke a larger number of cigarettes. This suggests differences in motivations for using tobacco, which may stem from varying social pressures and behavioral patterns.

The perception of e-cigarettes as less harmful (31.1%) is also concerning and aligns with a global trend of their increasing popularity (18). The 2018 review by Binns et al. illustrates the rapid international adoption of e-cigarettes by adolescents and its associated health risks. The authors highlight that by 2015, e-cigarettes had already become the most commonly used tobacco product among high school students in the United States, with 16% identifying as active users. The same review also points to evidence of direct harm, citing a large-scale study of 44,662 12-year-old students in Hong Kong that linked e-cigarette use to increased respiratory symptoms (adjusted odds ratio [aOR] = 1.39) (20). Furthermore, a 2021 systematic review by Bourke et al., which included studies with sample sizes varied from 13 to 44,462 from the US, Canada, Switzerland, and Hong Kong, found that coughing was one of the most common negative symptoms reported by adolescents upon initiating e-cigarette use. This places our local observations within the context of an international public health problem related to novel nicotine products (21).

A concerning finding is that a large percentage of students (31.1%) believed that electronic cigarettes are less harmful than traditional ones, indicating a significant gap in health education. Another study conducted in 2021 showed that coughing was a symptom reported by adolescents after starting to use e-cigarettes (21). Considering that e-cigarettes can be a gateway to traditional smoking and nicotine addiction, it is necessary to correct this belief by providing reliable information about health risks.

The issue of secondhand smoke (SHS) exposure in homes, which affected 42.4% of students in our study, also warrants international comparison. According to estimates presented in the 2021 review by Been et al., approximately 12% of children in the European Union are regularly exposed to SHS at home (22). This suggests the situation in the surveyed Polish region is considerably more serious than the EU average. Meanwhile, global data from the 2024 study by Flor et al. show that about 37% of the world’s population is exposed to SHS, with the problem being more pronounced in low- and middle-income countries (17). Our findings are therefore slightly above the global average. At the same time, the rate in the Chilean study was even higher at 62.3%, illustrating the varying scale of the problem across different socioeconomic settings (19).

Social factors also influence smoking. Nearly 40% of respondents received cigarettes from friends or older peers, and some managed to purchase tobacco products themselves despite legal restrictions. This highlights the need to strengthen the enforcement of the ban on selling tobacco products to minors and to conduct social campaigns aimed at changing group norms that promote smoking. Measures such as raising the legal age for purchasing tobacco products and reducing the number of sales points could help curb the tobacco epidemic among youth (22). Peer influence, identified in our research as a key factor in tobacco initiation, is also indicated as a significant predictor of other problematic health behaviors among Polish youth, including orthorexic tendencies (23, 24). This phenomenon underscores that social pressure and the patterns promoted within peer groups and on social media are a common denominator for various health risks during adolescence.

### 4.1 Strengths and limitations of the study

This study has several strengths. It utilized a large, randomly selected sample of adolescents from a specific, under-researched region of Poland, providing valuable local data for public health initiatives. The use of a standardized and internationally recognized questionnaire (GYTS) enhances the reliability of our findings and allows for comparability with national and international studies. Furthermore, the study’s scope was comprehensive, assessing not only traditional cigarettes but also e-cigarettes and other novel tobacco products.

However, the study is not without limitations. First, the lack of a formal sample size calculation may impact the generalizability of the results to the entire adolescent population of the voivodeship. Second, the data are self-reported, making them susceptible to social desirability and recall bias, which could lead to an underestimation of the true prevalence of smoking. Third, the cross-sectional design allows for the identification of associations but does not permit conclusions about causality. Finally, the data were collected in 2019, prior to the full implementation of the EU-wide ban on menthol cigarettes (May 2020) and the COVID-19 pandemic. These events may have since altered adolescent smoking behaviors, potentially limiting the applicability of our findings to the current context. The absence of logistic regression or odds ratio estimation limits the ability to quantify associations; however, the large, randomly selected sample still provides reliable prevalence estimates that remain informative for regional policy.

## 5 Conclusion

The phenomenon of cigarette and other tobacco product use among children and adolescents in the Subcarpathian Voivodeship is becoming increasingly widespread, despite growing public awareness of its harmful effects. Young people often turn to smoking due to curiosity, peer influence, and cultural or social norms. Despite knowledge of the adverse health consequences, many students continue to experiment with smoking, indicating a gap in the effectiveness of current educational efforts. The primary goal of this study was descriptive surveillance of adolescent tobacco use to inform local prevention efforts, not hypothesis testing or causal inference.

To counteract this phenomenon, more intensive preventive measures are necessary, involving both parents and children. Education should not only convey information about the harms of smoking but also foster healthy attitudes toward tobacco use. Parents should play an active role in raising their children’s awareness of the risks associated with smoking. Additionally, promoting alternative, substance-free leisure activities is crucial. Prevention efforts should be multifaceted, addressing various community levels to effectively curb the rising number of young smokers in the region.

## Data Availability

The raw data supporting the conclusions of this article will be made available by the authors, without undue reservation.
